# American Journal of Pharmacy

**Published:** 1856-12

**Authors:** 


*American Journal of Pharmacy*, published by authority of the Philadelphia College of Pharmacy. Edited by WM. PROCTOR, Jr. Professor of Pharmacy, &c.

The November number of this well-established bi-monthly journal has been received, and its contents are as varied and interesting as usual. It is well worthy of the patronage of both physicians and druggists.

Price, three dollars per annum.

